# Inherited privilege? First vs. continuing-generation medical students in Egypt, academic performance, extracurricular training and expectations: a cross-sectional study

**DOI:** 10.1186/s12909-024-06227-y

**Published:** 2024-11-06

**Authors:** Ayten Aboudeif, Youssef Elaraby, Mohamed Hany, Sara Nasser, Nadine Refaat, Yara Gamal Mohamed, Reem Youssry Khalil, Hashim Khabiry, Fatma Hussein Raafat, Nour Ghazy, Dina N.K. Boulos, Mostafa Yosef

**Affiliations:** 1 School of Medicine, Newgiza University, Giza, Egypt; 2 Department of Public Health, School of Medicine, Newgiza University, Giza, Egypt; 3 Department of Community, Environmental and Occupational Medicine, Faculty of Medicine, Ain Shams University, Cairo, Egypt

**Keywords:** First-generation medical students (FGMS), Continuing-generation medical students (CGMS), Egypt, Academic performance, Extracurricular training opportunities, Future expectations

## Abstract

**Background:**

The transition to college life is a highly demanding period for all students, especially when enrolling into an academically-challenging field as that of medicine. First-generation medical students represent an overlooked demographic and are thought to be more vulnerable to the stressors of college. This study’s aim is to explore the differences between first and continuing-generation medical students in Egypt regarding their academic performance, extracurricular training opportunities and future expectations.

**Methods:**

Two identical 24-item online questionnaires, in English and Arabic were distributed with five main themes: general demographics, academic performance, extracurricular training opportunities (outpatient clinics, hospitals and operating rooms), future expectations and if participants have relatives in the medical field.

**Results:**

Responses from 1652 eligible respondents from all 36 medical universities in Egypt were received. Continuing-generation medical students achieved a higher cumulative grade, with 46.1% getting an “Excellent” grade compared to only 38.4% in the first-generation. The gap between the two groups was best noted in Year 1 **(p-value < 0.001)**, as continuing-generation medical students were almost twice more likely to score an “Excellent” grade **[Odds Ratio = 1.85 (1.48–2.31)]**. First-generation group had less training opportunities in clinics (**p < 0.001**) and operating rooms (**p < 0.001**). There was a significant difference (**p < 0.001**) across all three clinical settings in regard to how the training opportunity was acquired. The majority of continuing-generation medical students credited their family members for it. Amongst those who wish to travel, the majority of the first-generation medical students plan to pursue their career in the United States; whereas, most of continuing-generation medical students aimed for the Middle East and Western Europe.

**Conclusion:**

First-generation medical students had lower grades at first mainly due to their lack of awareness of proper resources that were provided by the families of continuing-generation medical students. However, the challenges posed to first-generation medical students prompted the development of higher resilience, enabling them to catch up and even surpass their continuing-generation counterparts. Continuing-generation medical students obtained further extracurricular training (outpatient clinics and operating rooms), accessed mainly through their family members and connections; whereas, first-generation medical students received most of their training by searching for it themselves.

**Supplementary Information:**

The online version contains supplementary material available at 10.1186/s12909-024-06227-y.

## Background

### First generation medical students

For ages, college life has presented numerous obstacles and challenges to students, owing to the immense load they frequently encounter academically, emotionally, and financially. Some students may be unable to endure the aforementioned hardships, prompting them to give up and drop out. Confronting these issues should be a top priority as high quality education has been addressed by both the UN’s Sustainable Development Goals (SDGs) and Egypt’s vision 2030 Sustainable Development Strategy (SDS) to ensure that every student achieves their full academic potential [1, 2].

Noticeably, first-generation college students (FGCS) (who have no parent or legal guardian with a bachelor’s degree or higher) are even more vulnerable to these difficulties. They represent a large, often overlooked demographic even though a third of all college students in the United States are composed of FGCS [3]. A previous study revealed a gap between first and continuing-generation college students (CGCS) by demonstrating that FGCS have lower emotional intelligence levels when compared to their continuing-generation counterparts despite FGCS having higher resilience levels [4].

This situation is similar for medical students as the level of stress they encounter greatly surpasses what students of other majors face. For medical students in the US, the four-year graduation rate ranged from 81.6 to 84.3%; thus, up to 18% of those students had to graduate in 5–6 years or simply drop out [5].

In this study, a first-generation medical student (FGMS) will be defined as someone who is the first in their family to enter medical school. FGMS may be unfamiliar with the various studying methods and elements of medical school. For instance, they may be unaware of important learning materials and resources helpful in medical schools. Moreover, FGMS may be deprived of numerous extracurricular training opportunities that continuing-generation medical student (CGMS) may have because of their relatives. Further challenges that FGMS could encounter during their medical school years are: difficulties with studying and time management, feelings of imposter syndrome, and the fear of failure [6].

The difficulties that first-generation medical students face are not limited to academic performance; they also have a serious impact on their psychological health and their quality of life (QOL). As indicated in an Association of American Medical Colleges (AAMC) QOL survey carried out in 2013, results of Medical Student Life Survey (MSLS), which targeted medical school students and their personal wellbeing, showed that first-generation students scored worse than all other participants across all the well-being measures (higher stress and fatigue, and lower social support and quality of life) [7].

On the other hand, confronting the aforementioned obstacles is thought to have caused their heightened resilience, enabling them to be better adapted in the management of the high pressure and dynamic working environment [4].

### Egypt

When it comes to Egypt, a severe shortage of doctors was noted despite the great number of physicians who graduate from medical schools. As of the academic year 2020/2021, Egypt has had an estimated total number of 70,000 medical students [8, 9] Moreover, with Egypt possessing one of the oldest medical education establishments in the Middle East and North Africa (MENA) region and having 31 medical colleges registered in the World Directory of Medical Universities, as of 2022; it is surprising that WHO’s Global Health Workforce Statistics revealed that Egypt’s ‘Physicians per 1,000 people’ has a value of 0.7, a value much lower than the world’s 1.8 and MENA’s 1.4, let al.one the US’s value of 2.6 and the UK’s 5.8 [9, 10].

This emphasizes that the Egyptian health sector is under severe strain, which extends to the medical students. Several Egyptian studies showed that a significant number of their medical students’ participants suffered from stress, depression, and burnout [11, 12].

With limited research around the world, and none in Egypt, we aspire to bridge the critical knowledge gap on the next generation of physicians. We will shed light on the potential dissimilarities between first and continuing-generation medical students in Egypt, concerning their academic performance, extracurricular training opportunities and future expectations. This research, the first of its kind in Egypt, will serve as a starter study that may not only clarify whether these dissimilarities are indeed true, but will also act as a keystone for future research.

## Methods

### Definition of first and continuing-generation medical students

We decided that, for the purpose of this study, a first-generation medical student (FGMS) will be defined as someone who is the first in their family to enter medical school, whereas a continuing-generation medical student (CGMS) will be defined as someone who comes from a family in which a parent or a relative is a physician [13].

### Study design

The study aimed to assess whether there are differences between first-generation medical students and continuing-generation medical students in regard to academic performance, extracurricular training opportunities and future expectations. This cross-sectional study was conducted in Egypt via an online survey collected by convenience sampling. Taking into consideration the limited public platforms connecting medical students in Egypt, an online survey was the optimal tool to be used.

### Questionnaire and distribution

The items in the questionnaire comprised a mixture of question styles including multiple choice questions and matrices. Two identical 24-item questionnaires, in English and Arabic were distributed with five main themes:

1. General demographics.
2. Annual grades (for each previous year).
3. Whether participants have relatives in the medical field or not.
4. Extracurricular medical training (not included in current medical college program), if any and how they were acquired.
5. Participants’ expectations of their future salary and desired region to work in.

The full English Questionnaire is presented in supplementary material (1).

Clinical opportunities were assessed in terms of extracurricular training in 3 clinical settings: outpatient clinics, hospitals and operating rooms (OR), the extent of involvement was assessed (observation or hands-on participation) in each setting. The questionnaire was built using Microsoft form and distributed through social media channels (Facebook and WhatsApp) from the 1st of August 2022 till the 2nd of November 2022.

### Pilot study

The questionnaire was peer reviewed and a pilot study was carried out on 8 students from the school of Medicine at Newgiza University for clarity of questions’ wording, understandability, as well as the time needed to fill it. Both surveys (English and Arabic) were attempted during the pilot study. The results of the pilot study were not included in the final analysis.

Pilot results implied a clear discrepancy between FGMS and CGMS in the 3 parameters (grades, training opportunities and future expectations), but had multiple drawbacks. Respondents brought to our attention that some questions were redundant and that the initial draft took too long to complete. Repetitive revisions led to using branching logic in the survey design which led to more efficient data collection and provided a more user-friendly questionnaire.

### Participants

The study targeted current medical students in Egypt from both public and private universities due to the vastly different backgrounds, financially or socially. Egypt has 31 registered medical schools in the world directory of medical schools (WDOMS) as of August 2022. All academic years’ students in Egypt (1st year through 6th year) were eligible to participate. Non-Egyptian medical students studying in Egypt were excluded as they don’t face the same circumstances that Egyptian students face.

### Sample size

Using PASS 15 program for sample size calculation, it is estimated that a sample of 1000 first-generation and 500 continuing-generation medical students can detect even a small effect size difference between the two groups regarding qualitative outcome measures (academic grade) (Cohen’s h coefficient = 0.2) with power 99% and alpha error 0.05.

### Data analysis

The collected data was analyzed using IBM SPSS Statistics 27. Differences between first and continuing-generation medical students regarding grades, opportunities and future expectations were tested for significance using the Chi-Square test. *p*-value less than 0.05 was considered statistically significant for all tests. Cumulative grade was calculated by taking the average of the 5 years’ grades and rounding it to the nearest whole grade (Excellent ≥ 85%, Very good ≥ 75%, good ≥ 65%, pass ≥ 50% and fail < 50%), which is the established grading system in Egypt.

## Results

### Cohort demographics

Upon applying the inclusion and exclusion criteria onto a total of 1856 responses, 1652 were eligible respondents (Egyptian Medical students), 67.4%(*n* = 1114) were first-generation medical students (FGMS) and 32.6% (*n* = 538) were continuing-generation medical students (CGMS). Responses of 154 medical students were excluded from the Opportunities subsection due to a discrepancy in answering questions 17 and 18 in the survey. Responses were collected from 36 medical schools across Egypt, 82.6% (*n* = 1364) of responses came from public universities while 17.4% (*n* = 288) of responses came from private universities. The majority of respondents were from Year 6, 21.7% (*n* = 358), while the minority of students were from Year 1, 8.2% (*n* = 137). Mean age of respondents was 21.42 ± 2.21 and of the total eligible respondents, 758 (45.9%) were male and 894 (54.1%) were female.

**Table 1:** Percentage of medical students achieving each cumulative grade in both groups

	FGMS	CGMS
Fail	0.2% (*n* = 2)	0.6% (*n* = 3)
Pass	3.7% (*n* = 38)	3.9% (*n* = 19)
Good	18.6% (*n* = 191)	19.1% (*n* = 93)
Very Good	39.2% (*n* = 403)	30.2% (*n* = 147)
Excellent	38.4% (*n* = 395)	46.1% (*n* = 224)

Pearson Chi-Square **p-value = 0.008**. The significance was due to differences in “Very Good” and “Excellent” grades

### Grades

There was a significant difference in cumulative grade between the first and continuing-generation medical **students (p-value = 0.008)**. The significance was due to differences in “Very Good” and “Excellent” grades.

The most frequent cumulative grade obtained by each generation was “Very Good” in FGMS [39.2% (*n* = 403)], and “Excellent” in CGMS [46.1% (*n* = 224)]. Moreover, the percentage of CGMS having “Excellent” as cumulative grade was 7.7% higher than the percentage of FGMS having “Excellent” as cumulative grade 38.4% (*n* = 395). (Table 1)

Only the first-year score exhibited a significant difference between the two groups as the percentage of CGMS scoring “Excellent” grade [44.9% (*n* = 218)] was higher than the percentage of FGMS achieving “Excellent” grade [30.5% (*n* = 314)] with an **odds ratio (95% CI) of 1.85 (1.48–2.31) (p < 0.001)**. This difference in Year 1 grades was the main contributor to the significant differences of “Excellent” grade percentages seen in the cumulative grade between both groups. (Tables 1 and 2)

**Table 2:** “Excellent” grade & other grades in each year between FGMS & CGMS

	Grade	FGMS	CGMS	*p*-value	Odds Ratio (95% CI)
Year 1	Other grades Excellent	69.5% (*n* = 715) 30.5% (*n* = 314)	55.1% (*n* = 268) 44.9% (*n* = 218)	**< 0.001**	**1.85 (1.48–2.31)**
Year 2	Other grades Excellent	61.6% (*n* = 514) 38.4% (*n* = 320)	57.9% (*n* = 223) 42.1% (*n* = 162)	0.218	1.17 (0.91–1.49)
Year 3	Other grades Excellent	54.4% (*n* = 360) 45.6% (*n* = 302)	55.4% (*n* = 164) 44.6% (*n* = 132)	0.768	0.96 (0.73–1.26)
Year 4	Other grades Excellent	47.9% (*n* = 226) 52.1% (*n* = 246)	52.6% (*n* = 101) 47.4% (*n* = 91)	0.270	0.83 (0.59–1.16)
Year 5	Other grades Excellent	46.6% (*n* = 124) 53.4% (*n* = 142)	44.6% (*n* = 41) 55.4% (*n* = 51)	0.734	1.09 (0.67–1.75)

**Fig. 1:**
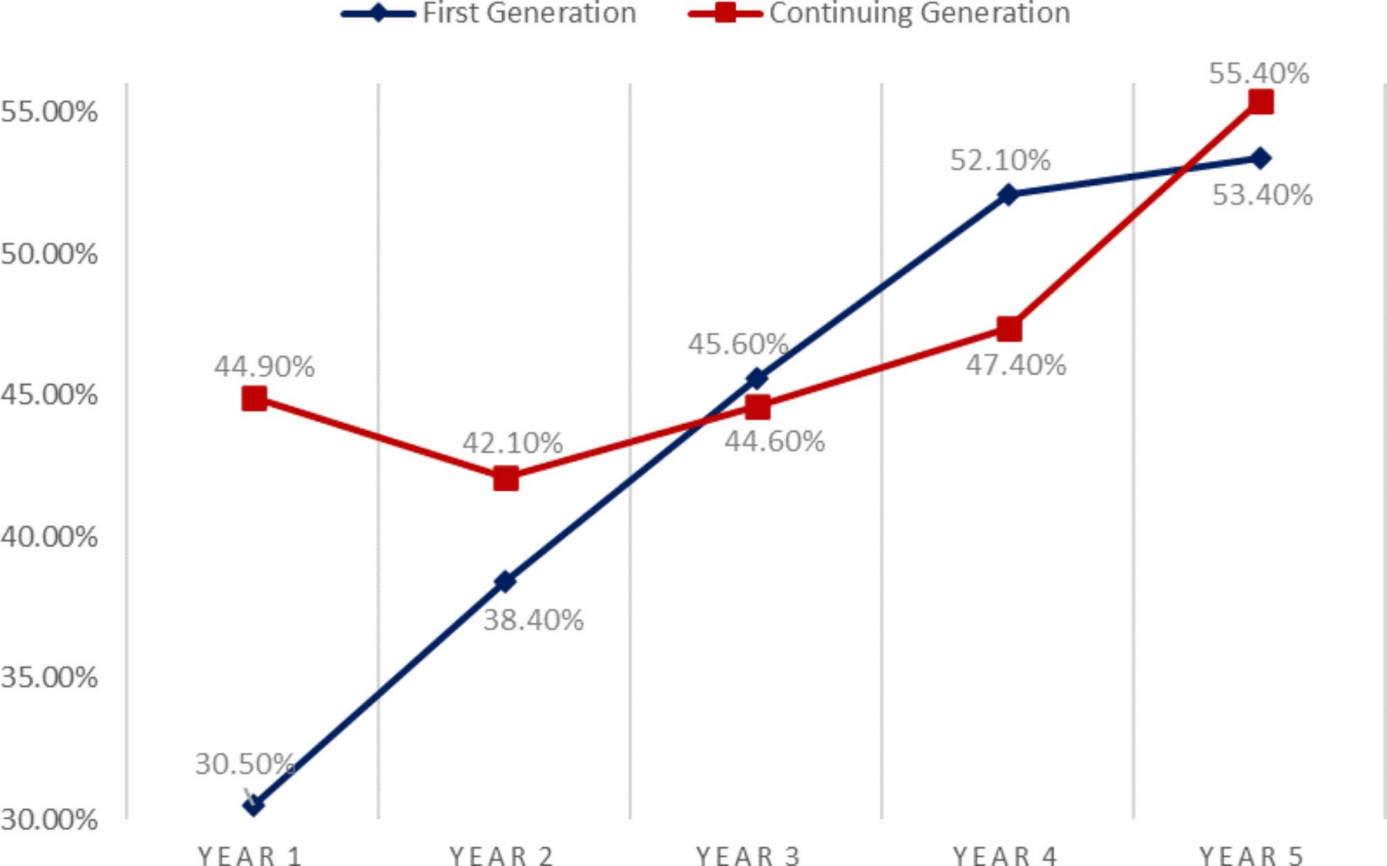
Percentages of “Excellent” grade in each year between FGMS & CGMS

An apparent trend was observed in the percentages of “Excellent” grades along the years between the two groups. Both groups had a progressive increase in the percentages of students achieving “Excellent” grades along the years. For CGMS, it was a slow fluctuating increase with a head start in front of FGMS in Year 1 (44.9% in CGMS vs. 30.5% in FGMS). However, FGMS had a steep constant increase; where they were achieved similar grades to CGMS in Year 3 (45.6% in FGMS vs. 44.6% in CGMS), surpassed them in Year 4 (52.1% in FGMS vs. 47.4% in CGMS), and almost having the same percentages in Year 5 (53.4% in FGMS vs. 55.4% in CGMS). (Fig. 1)

### Opportunities

#### Outpatient clinics & hospitals

CGMS had better opportunities in both observation and hands-on participation **(p < 0.001)** in outpatient clinics. For observations, CGMS were 18.0% (*n* = 88) vs. 10.0% (*n* = 101) in FGMS and for hands-on participation, CGMS were 6.7% (*n* = 33) vs. 3.1% (*n* = 31) in FGMS. However, upon comparing the hospital setting, there was no statistically significant difference (*p* = 0.455). (Table 3)

#### Operating rooms

CGMS had more opportunities in both observation and hands-on participation **(p < 0.001)** in operating rooms. For observations, CGMS were 22.7% (*n* = 111) vs. 16.7% (*n* = 169) in FGMS and for hands-on participation, CGMS were 4.9% (*n* = 24) vs. 1.8% (*n* = 18) in FGMS. (Table 3)

**Table 3:** Participation in each clinical setting between FGMS & CGMS

		None		Observation		Hands-on	*p*-value		
Outpatient Clinic	FGMS	86.9% (*n* = 877)		10.0% (*n* = 101)		3.1% (*n* = 31)	**< 0.001**		
	CGMS	75.3% (*n* = 368)		18.0% (*n* = 88)		6.7% (*n* = 33)			
Hospital	FGMS	69.0% (*n* = 696)		22.1% (*n* = 223)		8.9% (*n* = 90)	**0.455**		
	CGMS	66.1% (*n* = 323)		24.9% (*n* = 122)		9.0% (*n* = 44)			
Operating Room	FGMS	81.5% (*n* = 822)		16.7% (*n* = 169)		1.8% (*n* = 18)	**< 0.001**		
	CGMS	72.4% (*n* = 354)		22.7% (*n* = 111)		4.9% (*n* = 24)			

When assessing how the clinical opportunity was accessed, in all three clinical settings, most FGMS accessed the opportunity by their own search. Moreover, CGMS had a much higher percentage of accessing the opportunity through family members than FGMS in the 3 clinical settings; in outpatient clinics, 49.6% (*n* = 60) of CGMS vs. 7.6% (*n* = 10) of FGMS **(p < 0.001)**, in hospital settings 25.3% (*n* = 42) of CGMS vs. 1.9% (*n* = 6) of FGMS **(p < 0.001)** and in OR 34.1% (*n* = 46) of CGMS vs. 1.1% (*n* = 2) of FGMS **(p < 0.001)**. (Table 4)

**Table 4:** How the clinical opportunity was accessed between FGMS & CGMS

			FGMS		CGMS			*p*-value	
Outpatient Clinic	Family Member Family Friend		7.6% (*n* = 10) 21.2% (*n* = 28)		49.6% (*n* = 60) 7.4% (*n* = 9)			**< 0.001**	
	Own Search		71.2% (*n* = 94)		43.0% (*n* = 52)				
Hospital	Family Member Family Friend		1.9% (*n* = 6) 8.9% (*n* = 28)		25.3% (*n* = 42) 6.6% (*n* = 11)			**< 0.001**	
	Own Search		89.1% (*n* = 279)		68.1% (*n* = 113)				
Operating Theatre	Family Member Family Friend		1.1% (*n* = 2) 15.5% (*n* = 29)		34.1% (*n* = 46) 9.6% (*n* = 13)			**< 0.001**	
	Own Search		83.4% (*n* = 156)		56.3% (*n* = 76)				

### Future expectations

Regarding the expected future salary, no significant differences were noted between the 2 groups (*p* = 0.195). However, it is worth noting that the majority of both groups expect that their future salary to be either insufficient or just sufficient and a small percentage [8.1% (*n* = 90) in FGMS & 10.8% (*n* = 58) in CGMS] expect it to be more than sufficient.

When asked whether they plan on pursuing their career in Egypt or abroad, no significant difference was found between the two groups (*p* = 0.075); as 50.7% (*n* = 565) of FGMS wanted to travel abroad vs. 55.4% (*n* = 298) of CGMS. Upon calculating the total of all respondents who want to travel abroad in both groups, 52.2% (*n* = 863) wanted to continue abroad.

Nevertheless, amongst those students who intend to travel abroad, a significant difference was noted between the region they plan to work in **(p = 0.017)**, the significance was due to differences in choosing the US and Western Europe. The majority of FGMS [31.7% (*n* = 179)] chose the US, followed by the choice of the Middle East [(24.2% (*n* = 137)], while the majority of CGMS chose equally between the Middle East [(29.2% (*n* = 87)], and Western Europe [(29.2% (*n* = 87)]. (Table 5)

**Table 5:** FGMS & CGMS desired work abroad destination

	FGMS	CGMS
Middle East	24.2% (*n* = 137)	29.2% (*n* = 87)
Eastern Europe	4.8% (*n* = 27)	4.7% (*n* = 14)
Western Europe	22.1% (*n* = 125)	29.2% (*n* = 87)
USA	31.7% (*n* = 179)	24.5% (*n* = 73)
Other	17.2% (*n* = 97)	12.4% (*n* = 37)

Pearson Chi-Square **p-value = 0.017**. The significance was due to differences in choosing the US and Western Europe

## Discussion

The study aimed to detect the difference between FGMS & CGMS in “Excellent” grade rate throughout the years. The difference in “Excellent” grade between the two groups was most noticeable in Year 1. Another unexpected finding was the difference in the extracurricular training opportunities and how they were accessible.

### Grades

The differences in “Excellent” grades seen in the first year could possibly be attributed to the probability that FGMS may enroll into medical school with unrealistic expectations, not realizing the need to change their simpler methods of studying in high school into the demanding methods of studying the subjects of medicine. In contrast, CGMS are provided guidance on the complexities of medical school and have more realistic expectations.

Another probable cause of this gap may be linked to various benefits more readily available to CGMS, such as having better knowledge about the medical academic system and necessary studying resources (medical textbooks, online videos, and tutorials, etc.). Such invaluable tools are underutilized by the academically-naive FGMS, perhaps due to being unaware of their existence, underestimating their importance, or being unable to select the most beneficial and relevant resources to their academic stage. In addition, the CGMS are directly assisted by their family members whenever they encounter obstacles with studying.

As illustrated in Fig. 1, the difference in “Excellent” grades in both groups is clearly diminishing along the years with a steady and sustained rate, obliterating any major differences seen in first academic years. Our leading suggestion is that due to hardships experienced in the foundation years, FGMS have already gained enough experience and adaptability to perform on an equal footing with their continuing-generation counterparts, giving them greater resilience and flexibility than their peers. Two further investigations conducted in the United States in 2017 and 2021 reached similar conclusions [4, 6].

Year 4 often marks the initiation of the clinical years of medicine in most Egyptian universities, which may explain why more FGMS scored an “Excellent” grade compared to CGMS [14]. As clinical experience, undoubtedly, differs from the previous foundation years, our study suggests that the reason behind the grade turnover was the developed adaptability of FGMS, which allowed them to adapt and adjust to the perplexity of the experience quicker.

### Opportunities

A LinkedIn study examined how connections in online social networks impact job search results. Users’ strong links were especially useful in producing employment leads, interviews, and offers, whereas weak ties were ineffective in generating positive outcomes [15]. The medical field is no exception to this phenomena as nepotism is prevalent in medicine throughout the world. An example of this is expressed by an article in Greece, suggested that a continuing-generation medical student may be co-author in over 200 publications at a young age due to “support” from the entire school’s faculty in hopes of gratuity from the student’s parent [16].

Our findings were consistent in both outpatient clinics and operating room (OR). Even though most clinics in Egypt do not offer training programs affiliated with medical universities, numerous medical professionals train members of their immediate families in their own clinics to ease the transition of “inheriting” the family clinic. Similarly, in OR Training setting, many surgeons allow their medical relatives to attend and sometimes even participate in operating rooms, so that they get the chance to view procedures and gain early firsthand experience.

On the other hand, our analysis of hospital training revealed no statistically significant difference between the two generations. It is presumed that this is because many hospitals in Egypt adhere to a higher standard of professionalism regarding their training programs, making them accessible to all medical students regardless of their parentage. Furthermore, both groups routinely attend various hospital visits during their clinical years in their current curriculum, possibly leading to a general disinterest in pursuing additional hospital visits for both groups.

With most CGMS citing family members as the reason behind opportunities in the three clinical settings, having a family member in the medical industry was identified as the primary cause of disparity between the two groups. To the contrary, FGMS still depended heavily on their own search to acquire the training and had to actively seek out training opportunities, requiring more time and effort. FGMS attempted to use family-friends as connections, which turned out to be a weaker tie compared to the familial ties of CGMS. Yet, they fell short to the amount of training received. These findings demonstrate that there is a lack of official routes for opportunities in Egypt and a heavy reliance of the Egyptian medical field on connections.

### Future expectations

As previously mentioned, WHO’s Global Health Workforce Statistics exhibited that Egypt’s ‘Physicians per 1,000 people’ has a value of 0.7, a value much lower than the world’s 1.8 [10]. The results of the future expectations in this study may explain some reasons behind such a low number.

In terms of salaries, the majority of both groups believe that their salaries won’t be more than sufficient. These findings came as no surprise given the current inflationary state of Egypt and the fact that physicians’ careers typically begin late [17].

These findings can also be explained by the latest trend of multiple job holdings. According to the Medscape 2022’s physician compensation report, 36% of physicians took on additional work to increase their income [18]. Physicians typically work at their private clinics, hospitals, and universities. With this in mind, medical students are inclined to believe that a single source of income will be insufficient to fulfill their future needs.

Analyzing whether the participants planned on pursuing their career in Egypt or abroad, it was alarming that more than half of our respondents (52.2%) in both groups plan on working abroad. It is well known that medical students in Egypt face a myriad of struggles, with the financial situation being the main driving factor, often resulting in them leaving the country, contributing to Egypt’s ever-growing brain drain problem [19, 20].

When asked about their abroad postgraduate employment goals, respondents revealed an unexpected tendency. The fact that the United States is the most desired destination by FGMS may be attributed to not having enough information, optimism, or simply personal preferences. Unlike CGMS, who may have firsthand advice from medical relatives, FGMS may not be aware of the enormous number of challenges in the path of obtaining an American medical license.

The CGMS’ most desired destinations (Western Europe and Middle East equally) could be highly influenced by their medical relatives’ experiences. The general medical council reported that the number of Egyptian doctors joining the United Kingdom medical system has tripled, with that being the case, it was expected to witness such answers [21].

For several reasons (cultural and bureaucratic), the Middle East, especially the Gulf area, was found to be a common destination for both generations. Young doctors find it appealing as it provides a work-life balance, sufficient income along with career development. Not to mention that they would be close and within the same home region. Although European nations are among the top destinations, some young doctors are starting to prefer the Saudi option [22].

For long, undergraduate universities in the USA have had first-generation centered educational and administrative programs to address the pressing challenges they face; the need for university-culture readiness and the lack of professional/social networks required in career decision making [13]. Recently, US medical schools started following the footsteps of other universities, which led to several strategies and efforts being launched recently as mentioned by the AAMC [23].

Internationally, several programs have been founded to address and treat the gaps between first and continuing-generation students in different fields. An inspiring example is the David Geffen School of Medicine at University of California Los Angeles (UCLA) establishing a First-Gen program in 2017, with mentorship, academic assistance, educational transitions and home identity, and community-building defining the four pillars of their program [13]. Such worldwide initiatives act as an alarm that medical schools in Egypt must develop creative solutions to maximize students’ full potential.

Based on the study findings, it is recommended that medical universities in Egypt initiate a program aimed at first-year medical students as well as high-school students interested in attending medical school. This program should include a detailed illustration of what medical school entails, such as: a clear tutorial of subjects taught in the foundation years of medicine, education about the Egyptian medical school system, different studying resources and supplementary material (question-banks and mock exams) that are appropriate for their academic stage.

Another program could be integrated for medical students of all years. One suggested program would inform them of the different specialties in medicine and their advantages and disadvantages along with detailed information of possible routes to each of the regions mentioned in our study. Furthermore, universities may implement programs to provide FGMS with critical extracurricular activities such as internships, elective modules, research opportunities, and clinical training.

A mentorship program that pairs First-generation alumni doctors in mentor roles with FGMS as their respective mentees would enable them to provide knowledge on how they overcame the hurdles that come with being a first-generation.

A nationwide initiative of student-led associations where FGMS engage and share their experiences with one another could garner a feeling of solidarity, raise morale, and help in eliminating the feeling of imposter syndrome.

Further studies are needed to determine whether or not implementing these initiatives is effective.

### Limitations

The use of non-random convenience sampling via social media platforms (Facebook and Whatsapp) resulted in a lack of generalizability in the sample. Another limitation of using an online survey is our inability to calculate the response rate. Thus, the number of students in the study is not an accurate representation of the total number of students in each university.

Thirdly, using an online survey makes the results liable to selection bias. To address this limitation, a large sample size was targeted. In addition, the survey was shared on different social media platforms to attempt equitable reach to the intended population.

Our data was gathered through self-reporting, which is prone to bias, especially when it comes to their academic performance; thereby, an anonymous questionnaire was provided.

## Conclusion

FGMS initially had relatively poorer grades than CGMS in Year 1, maybe due to their lack of awareness of proper resources that were provided by the families of CGMS. However, the challenges posed to FGMS most-likely prompted the development of greater resilience, enabling them to catch up with and even surpass their CGMS counterparts.

CGMS obtained further extracurricular training (outpatient clinics and operating rooms), accessed mainly through their family members and connections; whereas, FGMS received most of their training by searching for it on their own.

## Electronic supplementary material

Below is the link to the electronic supplementary material.

Supplementary Material 1

Supplementary Material 2

Supplementary Material 3

## Data Availability

The datasets used and/or analyzed during the current study are available from the corresponding author on reasonable request.

## Abbreviations

AAMC: Association of American Medical Colleges
CGCS: Continuing-generation college students
CGMS: Continuing-generation medical students
FGCS: First-generation college students
FGMS: First-generation medical students
MENA: Middle East & North Africa
MSLS: Medical Student Life Survey
OR: Operating Room
QOL: Quality of life
SDGs: Sustainable Development Goals
SDS: Sustainable Development Strategy
UCLA: University of California Los Angeles
US: United States
