# Supplementary material for: Inherited privilege? First vs. continuing-generation medical students in Egypt, academic performance, extracurricular training and expectations: a cross-sectional study
Source: BMC Med Educ. 2024 Nov 6;24:1274. doi: 10.1186/s12909-024-06227-y (PMC11542418; doi:10.1186/s12909-024-06227-y)
Supplement: Supplementary file 1 — Supplementary Material 1 [file 12909_2024_6227_MOESM1_ESM.pdf]

First vs Continuing Generation Medical Students Survey

* Required

1. I consent *

This questionnaire is designed to learn the problems that first generation medical students might face.
The answers are anonymous.

- Yes
- No

Demography

2. Nationality *

- ◯ Egyptian
- ◯ Non-Egyptian

3. Age *

____

The value must be a number

4. Sex *

- ◯ Male
- ◯ Female

5. Are you a medical student? (Human Medicine) *

- ◯ Yes
- ◯ No

6. University *

- ○ Ain Shams University
- ○ Al-Azhar University
- ○ Alexandria University
- ○ Armed Forces
- ○ Assiut University
- ○ Aswan University
- ○ Badr University
- ○ Benha University
- ○ Beni Suef University
- ○ Cairo University Kasr Alainy
- ○ Damietta University
- ○ Delta University for Science and Technology
- ○ Fayoum University
- ○ Galala University
- ○ Helwan University
- ○ Horus University

- Kafrelsheikh University
- King Salman International University
- Luxor University
- Mansoura University
- Menoufia University
- Merit University
- Minia University
- Misr University for Science and Technology
- Modern University for Technology and Information
- Nahda University
- New Valley University
- Newgiza University
- October 6 University
- Port Said University
- Sohag University
- South Valley University Qena
- Suez Canal University
- Suez University

- Tanta University
- Zagazig University

Academic Performance

7. What is your current academic year starting 2022-2023? *

- ◯ Year 1
- ◯ Year 2
- ◯ Year 3
- ◯ Year 4
- ◯ Year 5
- ◯ Year 6

8. What is your average grade for Year 5? *

- ◯ Excellent
- ◯ Very good
- ◯ Good
- ◯ Pass
- ◯ Fail

9. What is your average grade for Year 4? *

- Excellent
- Very good
- Good
- Pass
- Fail

10. What is your average grade for Year 3? *

- Excellent
- Very good
- Good
- Pass
- Fail

11. What is your average grade for Year 2? *

- ◯ Excellent
- ◯ Very good
- ◯ Good
- ◯ Pass
- ◯ Fail

12. What is your average grade for Year 1? *

- ◯ Excellent
- ◯ Very good
- ◯ Good
- ◯ Pass
- ◯ Fail

Information About Your Family

13. Do you have someone in the family in the medical field? *

- ◯ Yes
- ◯ No

14. Are they a first degree relative? (Father, Mother, Sibling) *

- ◯ Yes
- ◯ No

15. Are they a second degree relative? (Grandparent, Uncle, Aunt) *

- ◯ Yes
- ◯ No

16. Are they a third degree relative? (Cousin at the first level) *

- ◯ Yes
- ◯ No

Medical Training Outside of University Hours

17. In Egypt, were you provided any training opportunities in these fields outside of your university's teachings? *

Click on arrowhead for the list.

	None	Observatio n	Hands-On (participat ed/helped)
Clinic/Outpa tient	○	○	○
Hospital	○	○	○
Operating Room/Theat re	○	○	○

18. How was it provided? *

Click on arrowhead for the list.

	Not Received	My Search	A Family Member	A Family Friend
Clinic/Outpatient	○	○	○	○
Hospital	○	○	○	○
Operating Room/Theatre	○	○	○	○

Future Expectations

19. Do you expect your future salary to be: *

- ◯ Insufficient
- ◯ Sufficient
- ◯ More than Sufficient

20. Where do you expect/plan to work? *

- ◯ Egypt
- ◯ Abroad

21. Where do you want to work abroad? *

- ◯ USA
- ◯ Western Europe
- ◯ Eastern Europe
- ◯ Middle East
- ◯ Other

Other

This content is neither created nor endorsed by Microsoft. The data you submit will be sent to the form owner.

Microsoft Forms
