# Supplementary material for: Inherited privilege? First vs. continuing-generation medical students in Egypt, academic performance, extracurricular training and expectations: a cross-sectional study
Source: BMC Med Educ. 2024 Nov 6;24:1274. doi: 10.1186/s12909-024-06227-y (PMC11542418; doi:10.1186/s12909-024-06227-y)
Supplement: Supplementary file 2 — Supplementary Material 2 [file 12909_2024_6227_MOESM2_ESM.pdf]

Public vs Private Universities

Results:

82.6% (n=1364) of responses came from public universities while 17.4% (n=288) of responses came from private universities.

FGMS' responses in public universities were 961 (85.7%) vs 161 responses (14.3%) in private universities.

Cumulative grades of public universities' students portrayed a significant difference **(p <0.001)**. This significance was due to differences in "Very Good" and "Excellent" grades with 41% (n=359) of the FGMS scoring "Excellent", lower than CGMS [52% (n=191)], following the overall trend. While there was no significant difference in cumulative grades in private universities' students (p=0.885).

In public universities, FGMS acclaimed lower percentages of "Excellent" grades in each academic year, though only Year 1's grades were statistically significant. **(Figure 1)**. Significant differences in Year 1's "Excellent" grades **(p <0.001)** showed that the percentage of CGMS scoring "Excellent" grade [50.1% (n=184)] was higher than in FGMS [31.7% (n=278)] with **odds ratio (95% CI) of 2.16 (1.69-2.77)**, adhering to the overall trend.

In private universities, regarding each individual year, percentages of FGMS who got "Excellent" grades were less than percentages of CGMS in the first two years. However, unlike their peers in public universities, FGMS managed to pick up their grades and surpass CGMS in the three following years with a progressive increase in the gap. **(Figure 1)**.

Figure 1. “Excellent” grades between FGMS & CGMS in public & private universities

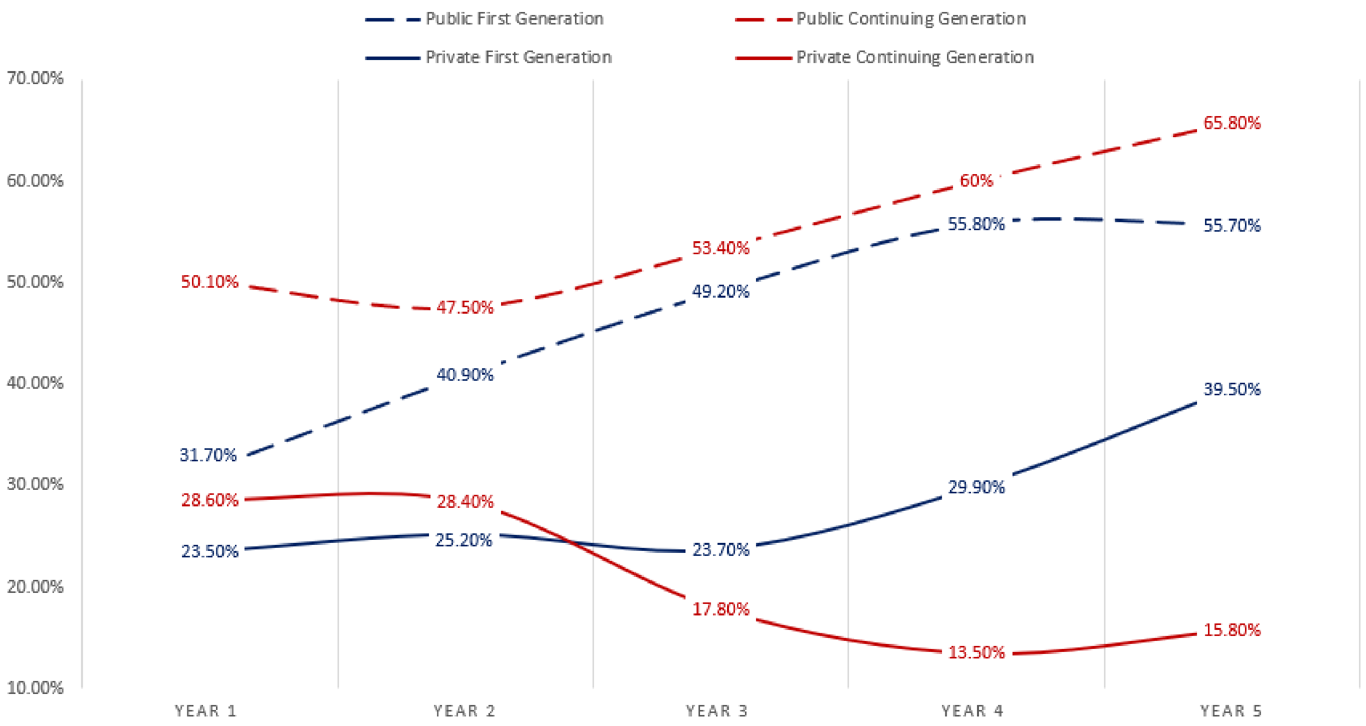

Only Year 4 had a significant difference **(p=0.034)** with percentages of FGMS achieving “Excellent” grades being 29.9% (n=20) which is more than the percentage of CGMS achieving “Excellent” grade 13.5% (n=7) with an **odds ratio (95% CI) of 2.74 (1.06-7.09)**.

Discussion:

The disparity in grades between first and continuing-generation medical students, whether overall or in individual years, was primarily due to differences witnessed at public universities. This could be explained by the fact that the vast majority of CGMS's parents/relatives are alumni of public universities, and, in many cases, are part of the working staff, making it easier for the parents to assist CGMS academically. This is not the case for many private universities, particularly those that are newer and are collaborating with universities abroad to provide a curriculum similar to theirs[15]. These relatively new systems can prove to be unfamiliar and foreign to both students and family members in the medical field, limiting the level of aid.

When students from the two groups are placed on equal footing, as in the uncharted academic systems of private universities, the resilience acquired by FGMS gives them the edge over their peers in the later years of medical school, best seen in Year 4 (**Figure 1**).

Despite the fact that it is outside the scope of our study, an unexpected phenomenon was observed; a clear disparity in grades between students at public and private universities, whether in first or continuing-generation students.

This could be due to the fact that public universities in Egypt have been committing to the same form of academic assessment for decades, while each private university holds its own methods of examinations. Another suggestion is that because the minimal acceptance grades in private universities are lower, private university students may be less academically capable than their peers in public universities.
