# Supplementary material for: Inherited privilege? First vs. continuing-generation medical students in Egypt, academic performance, extracurricular training and expectations: a cross-sectional study
Source: BMC Med Educ. 2024 Nov 6;24:1274. doi: 10.1186/s12909-024-06227-y (PMC11542418; doi:10.1186/s12909-024-06227-y)
Supplement: Supplementary file 3 — Supplementary Material 3 [file 12909_2024_6227_MOESM3_ESM.pdf]

University	Total Respondents (%)
Newgiza University	121 (7.32)
Ain Shams University	87 (5.27)
Al-Azhar University	126 (7.63)
Alexandria University	95 (5.75)
Assiut University	47 (2.85)
Aswan University	26 (1.57)
Armed Forces University	4 (0.24)
Badr University	2 (0.12)
Benha University	33 (2.00)
Beni Suef University	109 (6.60)
Cairo University	157 (9.50)
Damietta University	4 (0.24)
Delta University for Science and Technology	11 (0.67)
Fayoum University	33 (2.00)
Galala University	12 (0.73)
Helwan University	76 (4.60)
Horus University	1 (0.06)
Kafrelsheikh University	32 (1.94)
King Salman International University	6 (0.36)
Luxor University	5 (0.30)
Mansoura University	87 (5.27)
Menoufia University	65 (3.93)
Merit University	4 (0.24)
Minia University	57 (3.45)
Misr University for science and Technology	42 (2.54)
Modern University for Technology and Information	17 (1.03)
Nahda University	43 (2.60)
New Valley University	1 (0.06)
October 6 University	34 (2.06)
Port Said University	16 (0.97)
Sohag University	51 (3.09)
South Valley University	18 (1.09)
Suez Canal University	47 (2.85)
Suez University	7 (0.42)
Tanta University	91 (5.51)
Zagazig University	85 (5.15)
